# Bioengineered Co-culture of organoids to recapitulate host-microbe interactions

**DOI:** 10.1016/j.mtbio.2022.100345

**Published:** 2022-07-01

**Authors:** Min Beom Kim, Soonho Hwangbo, Sungho Jang, Yun Kee Jo

**Affiliations:** a Department of Biomedical Convergence Science and Technology, School of Convergence, Kyungpook National University, Daegu 41566, South Korea; b Department of Chemical Engineering, Gyeongsang National University, Jinju 52828, South Korea; c Department of Materials Engineering and Convergence Technology, Gyeongsang National University, Jinju 52828, South Korea; d Division of Bioengineering, College of Life Sciences and Bioengineering, Incheon National University, Incheon 22012, South Korea; e Department of Bioengineering and Nano-Bioengineering, Incheon National University, Incheon 22012, South Korea; f Research Center for Bio Materials & Process Development, Incheon National University, Incheon 22012, South Korea; g Cell and Matrix Research Institute, Kyungpook National University, Daegu 41944, South Korea

**Keywords:** Organoids, Co-culture model, Host-microbe interaction, Human physiology, Pathophysiology

## Abstract

The recent spike in the instances of complex physiological host-microbe interactions has raised the demand for developing *in vitro* models that recapitulate the microbial microenvironment in the human body. Organoids are steadily emerging as an *in vitro* culture system that closely mimics the structural, functional, and genetic features of complex human organs, particularly for better understanding host-microbe interactions. Recent advances in organoid culture technology have become new avenues for assessing the pathogenesis of symbiotic interactions, pathogen-induced infectious diseases, and various other diseases. The co-cultures of organoids with microbes have shown great promise in simulating host-microbe interactions with a high level of complexity for further advancement in related fields. In this review, we provide an overview of bioengineering approaches for microbe-co-cultured organoids. Latest developments in the applications of microbe-co-cultured organoids to study human physiology and pathophysiology are also highlighted. Further, an outlook on future research on bioengineered organoid co-cultures for various applications is presented.

## Graphical abstract

**Figure:**
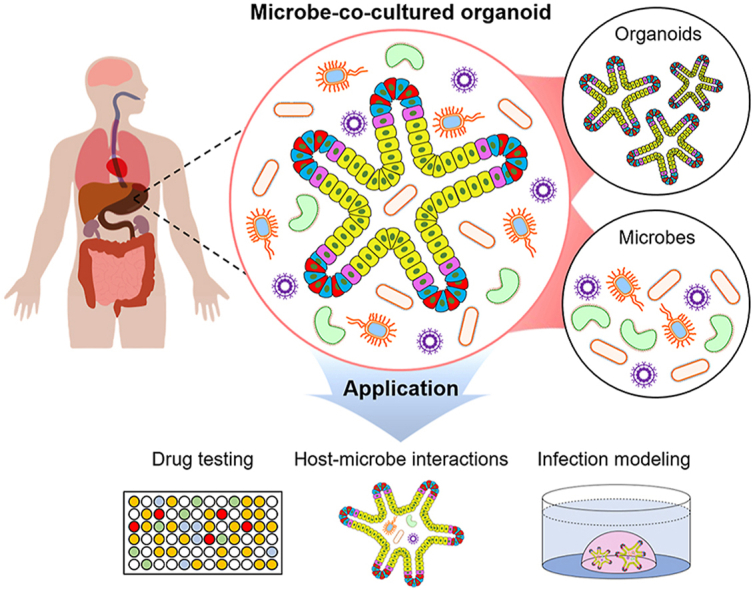

## 1 Introduction

Microbes have increasingly emerged as key players in modulating various physiological and pathophysiological processes in a human host [1,2]. The majority of microbes in extensively colonized tissues, including those of the gastrointestinal tract and upper respiratory tract, aid in normal development and function of human body system. While they protect the host from infection and maintain host health, some microbial components and the resulting changes in the environment can cause infectious diseases such as coronavirus disease 2019 (COVID-19) and several types of ulcerative [1,[3], [4], [5], [6]]. Despite numerous efforts in the last decade, research into diverse host-microbe interactions and their impact on the development and progression of diseases remains at nascent stages due to limited availability of human tissues and the lack of *in vitro* models [7].

Organoids are three-dimensional (3D) multicellular tissue constructs derived from the self-organization of stem cells [8]. Primary stem cells or pluripotent stem cells cultured *in vitro* under conditions similar to the specific microenvironment in the human body allow the generation of organoids that mimic their corresponding organs, such as the small intestine, brain, lung, and stomach [[9], [10], [11], [12]]. Conventional two-dimensional (2D) culture systems are relatively simple but pose challenges in recapitulating the composition of differentiated cells and tissue function [13]. The use of *in vivo* models can overcome these problems and permit the execution of more complex studies in the presence of interconnected cells and tissues. Among these, *in vivo* mouse models have been successfully adopted to study the host-microbe interactions owing to their genetic and taxonomic similarities to the human microbiome [14,15]. Nevertheless, animal models are associated with ethical issues, greater time and financial costs, and species-associated variance [16]. Organoids mimic the gene and protein expression, metabolic function, and microstructure of *in vivo* tissues of animal models, offering a promising *in vitro* system to address the gap between 2D cultures and *in vivo* animal experiments [17]. For example, upon infection with microorganism, the organoid models have shown higher microbial-associated proteins than the 2D culture model, directly highlighting the suitability and superiority of organoids for modeling of host-microbe interactions [18,19]. In particular, patient-derived organoids allow for the pathophysiological studies of the cellular mechanism and diversity, from the of host-microbe interactions viewpoint [20].

**Fig. 1:**
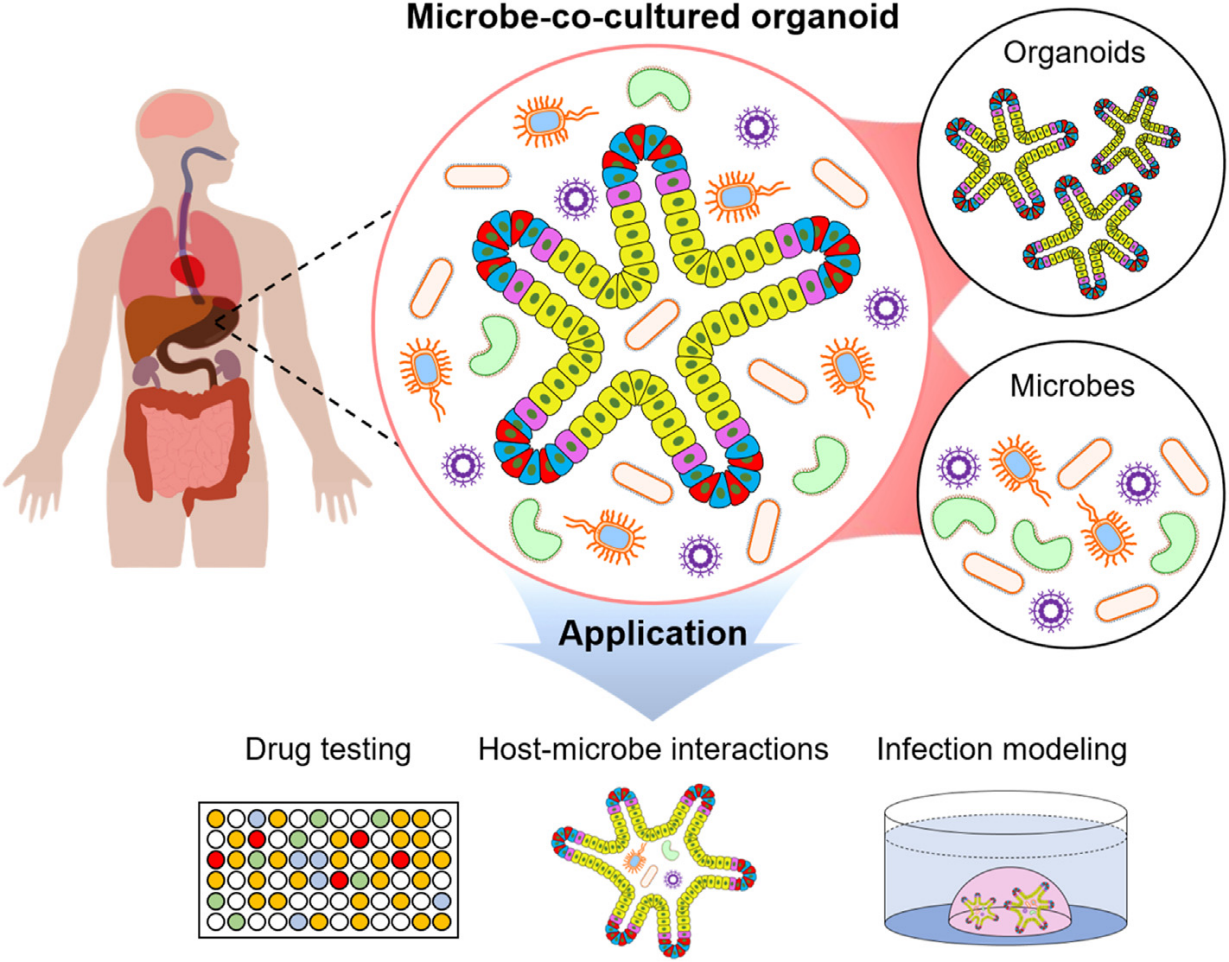
Schematic illustration for microbe-co-cultured organoid models and various applications.

To the best of our knowledge, this is the first review that provides a comprehensive overview of development in the bioengineering of organoids to study the host commensal/pathogenic microbial interactions, in an organ-specific manner (Fig. 1). We have summarized the experimental techniques and key features of organoid co-cultures. Next, we describe recent breakthroughs in organoids co-cultures to recapitulate several target organs in the gastrointestinal, respiratory, nervous, reproductive, and urinary systems. Finally, we discuss the practical limitations of co-cultured organoids and offer some perspectives and outlook for the future.

## 2 Preparation of organoids-microbial co-culture models

**Fig. 2:**
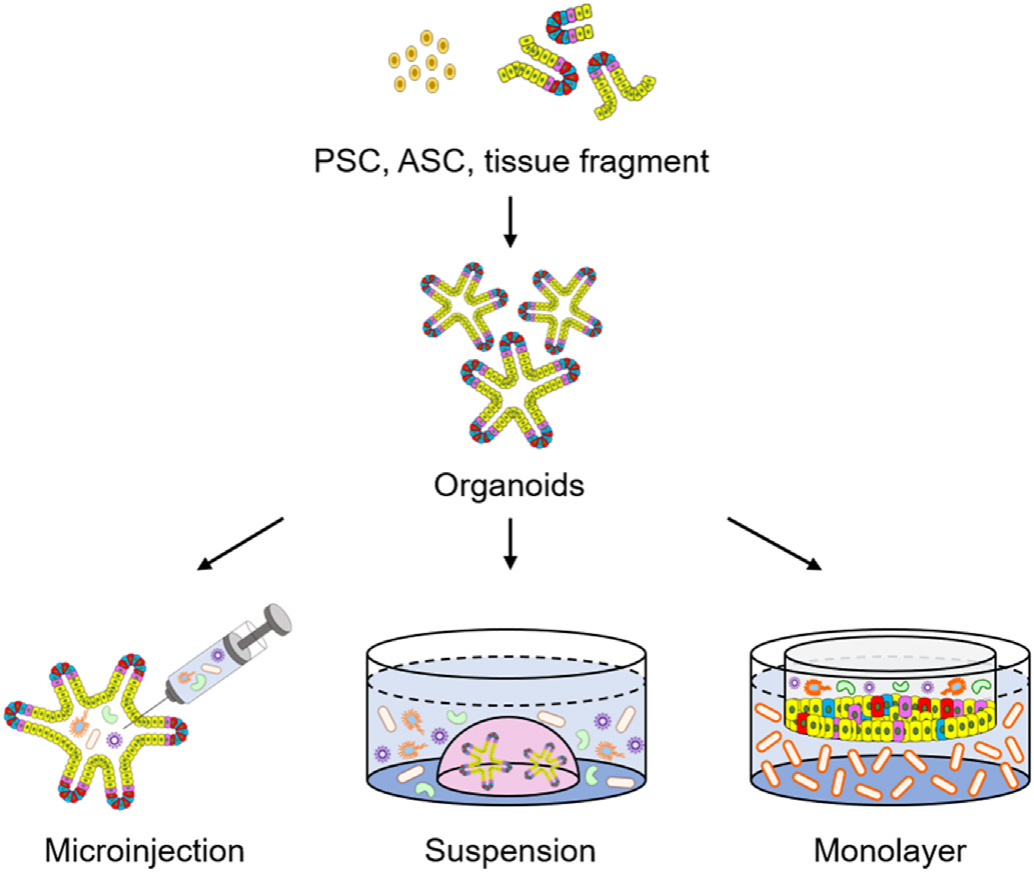
Overview of methods for constructing microbe-co-cultured organoids model.

To understand the interactions between organoids and microbes in greater depth, it is imperative to simulate an environment that mimics that of the human body harboring resident microbes. Particularly, most organoid culture models have an enclosed architecture surrounded by cells, thus limiting access and hindering the interaction between the inner apical epithelial cells and microorganisms. To overcome these limitations in 2D and 3D cultures, different approaches, such as the microinjection technique, generation of apical-out organoids and monolayer cultures, have been adopted (Fig. 2). In this section, we discuss methods for effectively exposing microbes to organoids.

### 2.1 Microinjection into the organoids

Microinjection has been widely used to mechanically deliver DNA, RNA, protein, peptide, sperms, and drugs to single cells [21]. Additionally, infectious diseases have been modeled through and intracellular injection of various microbes, such as *Salmonella* [22], *Clostridium difficile* [23], *Escherichia coli* [24], and viruses [25], to obtain mechanistic insights. Microinjection into the lumen of organoids facilitates the interaction of the microbes with the apical side of organoids and has been considered an accurate, efficient, and practical method of delivering microbes, with low cytotoxicity for the organoid-microbe co-culture [21,26]. In particular, since organoids have a lower oxygen concentration compared to the external environment, the use of intra-organoid microinjection in the co-culture of anaerobic microbes can help minimize their exposure to oxygen [27]. Microscale device capable of highly efficient and reproducible microinjection of microbes into the lumen of organoids has been developed [28]. Moreover, a double-barrel perfusion system has shown to provide constant luminal access and allow regulation of luminal contents and luminal mixing, thereby enabling the increased success rate of perfusion with a higher gastrointestinal motility and microbiome stability. However, manual microinjection restricts high-throughput experiments and often leads to variation in the co-cultured organoids generated [20,29,30]. The injection of varying number of microbial cells into the lumen can further exacerbate the non-uniformity in multiplicity of infection of organoids caused by different sizes and cell composition ratios [28,29]. In addition, the maintenance of oxygen/nutrient levels suitable for the symbiosis of organoids and microbes is a challenge that still remains to be solved [27,29,31].

### 2.2 Suspension on the outside of the organoids

Most organoids are generated as basal-out structures and can be facilely co-cultured with microbes in a simple suspension [[32], [33], [34], [35], [36]]. However, this approach limits the access of microbes to the apical side of organoids mainly due to their closed conformation. A simple suspension of microbes in the medium for the culture of apical-out organoids, in which the luminal and basal structures are inverted, can be an alternative method that maintains barrier integrity and functional properties by modulating extracellular matrix (ECM) proteins during cultivation [[37], [38], [39], [40]]. In particular, the apical-out organoids could address the limitations of conventional intra-organoid microinjection, such as multi-equipment requirement, labor-intensive process, and vulnerability to the influx of epithelial cells into the lumen. Nevertheless, on comparing with common basolateral-out organoids, the apical-out organoids still pose challenges due to their low proliferation, poorly controlled differentiation, and uncertainty in the complete reversal of inner and outer cavities [27,39].

### 2.3 Culture with organoids-derived monolayers

Organoid-derived monolayer cultures can be established by incubating single cells or organoid fragments dissociated enzymatically from 3D organoids on a Transwell or flat culture surface in a medium containing microbes, supplemented with several types of nutrients [[41], [42], [43], [44], [45], [46], [47]]. During the diffusion of the microbe-containing medium through the dome of complex matrices (*i.e*., Matrigel or Basement Membrane Extract), organoids located at the edge of the dome are more difficult to access by microbes than those located in the center of the dome. However, single cell monolayers derived from organoids can increase the access rate of microbes toward the cell lumen with an additional advantage of allowing easy sample collection at various time points [42]. In addition, organoid-derived monolayers can be employed as a powerful model for co-culture with aerobic bacteria, since the Transwell has a larger area in contact with oxygen than those with anaerobic bacteria [27,42]. To generate organoids with resident anaerobes, an anaerobic environment needs to be maintained with a continuous provision of nutrients; failure to do so shortens the lifespan of the organoids and microbes are induced to overgrow, making it difficult to achieve a stable, long-term culture.

To overcome the limitations of the different levels of oxygen requirements between epithelial cells and microbes in Transwell co-cultures, a 2D culture system for human colonic epithelium called the intestinal hemi-anaerobic co-culture system (IHACS) consisting of a hypoxic apical chamber and normoxic basal chamber has been developed [27,48,49]. A hypoxic environment is essential for anaerobic microbes, while a normoxic condition is critical for the survival of epithelial cells. Therefore, the IHACS system can complement the environmental gap between organoids and microbes to ensure their survival [48]. Besides, a variety of culture methods, including organoids-on-chip and a microfluidics-based human microbial crosstalk (HuMiX) model, have been extensively used to address the limitations of Transwell cultures [[50], [51], [52]].

## 3 Microbe-co-cultured organoids of different organs

Research into microbes and the physiological/pathological understanding of various organs that have characteristics similar to those of tissues and organoids of the human body is steadily progressing through the use of organoid models (Table 1). In this section, we discuss organ-specific co-culture systems with pathogens and symbionts to understand interactions and molecular mechanisms in the human body.

**Table 1:** Summary of co-cultured organoids developed for the study of host-microbe interactions in various organs. Abbreviation: Severe acute respiratory syndrom-coronavirus-2, SARS-CoV-2.

Organ	Origin	Model	Microbe	Ref
Intestine	Mouse	Microinjection	*Salmonella*	[64]
			*Cryptosporidium parvum*	[164]
		Suspension	Rotavirus	[19]
			Influenza virus	[33]
			Salmonella	[67]
			*Lactobacillus*	[73,76,77]
		Monolayer	*Toxoplasma gondii*	[84]
			*Giardia duodenalis*	[84]
	Human	Microinjection	*Salmonella*	[22,65,66]
			*Clostridium difficile*	[23,68]
			*Escherichia coli*	[24,28,74,78]
			*Lactobacillus*	[72]
		Suspension	Rotavirus	[59,60]
			Reovirus	[62]
			*Lactobacillus*	[75]
			*Escherichia coli*	[75]
		Monolayer	*Salmonella*	[44]
			Norovirus	[61]
			*Escherichia coli*	[79]
Colon	Mouse	Microinjection	*Salmonella*	[85]
	Human	Suspension	*Lactobacillus*	[89]
		Monolayer	*Bacteroides thetaiotaomicron*	[42]
			*Escherichia coli*	[43,47]
			*Bifidobacterium adolescentis*	[48]
			*Bacteroides fragilis*	[48]
			*Clostridium butyricum*	[48]
			*Akkermansia muciniphila*	[48]
Liver	Human	Suspension	Hepatitis B virus	[94,95]
		Monolayer	Hepatitis E virus	[92]
Stomach	Mouse	Microinjection	*Helicobacter pylori*	[100,101,103]
	Human	Microinjection	*Helicobacter pylori*	[98,99]
		Suspension	*Helicobacter pylori*	[102]
		Monolayer	SARS-CoV-2	[45]
			Epstein-Barr virus	[46]
Gallbladder	Mouse	Suspension	*Salmonella*	[35]
		Monolayer	*Salmonella*	[105]
Lung	Human	Microinjection	*Mycobacterium tuberculosis*	[108]
			*Mycobacterium abscessus*	[108]
			Human parainfluenza virus type 3	[111]
			Respiratory syncytial virus	[113]
			*Aspergillus fumigatus*	[126]
			*Cryptosporidium parvum*	[163,164]
		Suspension	Respiratory syncytial virus	[115]
			SARS-CoV-2	[118,119,122]
		Monolayer	Influenza A virus	[117]
			SARS-CoV-2	[120]
Brain	Human	Suspension	Zika virus	[25,34,128,129,131,132,135]
			Human immunodeficiency virus	[136]
			Herpes simplex virus	[137]
			*Toxoplasma gondii*	[139]
Fallopian tube	Mouse	Suspension	*Chlamydia trachomatis*	[145]
	Human	Monolayer	*Chlamydia trachomatis*	[140]
Cervix	Human	Suspension	*Chlamydia trachomatis*	[149]
Kidney	Human	Suspension	SARS-CoV-2	[18,36,155]
			BK-virus	[152]
		Monolayer	SARS-CoV-2	[154]
Blood vessel	Human	Suspension	SARS-CoV-2	[155]
Tonsil	Human	Suspension	SARS-CoV-2	[166]
Eye	Human	Suspension	SARS-CoV-2	[167]
Oral	Human	Suspension	Herpes simplex virus	[150]
			Human papillomavirus	[150]

### 3.1 Digestive system

#### 3.1.1 Intestine

Intestinal organoids are composed of various intestinal cell types, including epithelial cells, goblet cells, enteroendocrine cells, and Paneth cells, in addition to stem cells, and have been utilized as a 3D *in vitro* model to study the development and diseases of the intestinal epithelium [53]. The structural and functional similarities to real intestinal tissue render intestinal organoids ideal to model disease of the small intestinal, such as inflammatory bowel disease and colorectal cancer [54,55]. In addition, co-cultures of intestinal organoids with various pathogenic microbes, including *C. difficile*, *Salmonella*, and viruses, have been utilized as a research model for understanding viral pathogenesis and host-microbial dynamics. As the gut is more densely populated with microbes than other organs, many studies have previously focused on the co-culture of intestinal organoids with microbes [56,57].

**Fig. 3:**
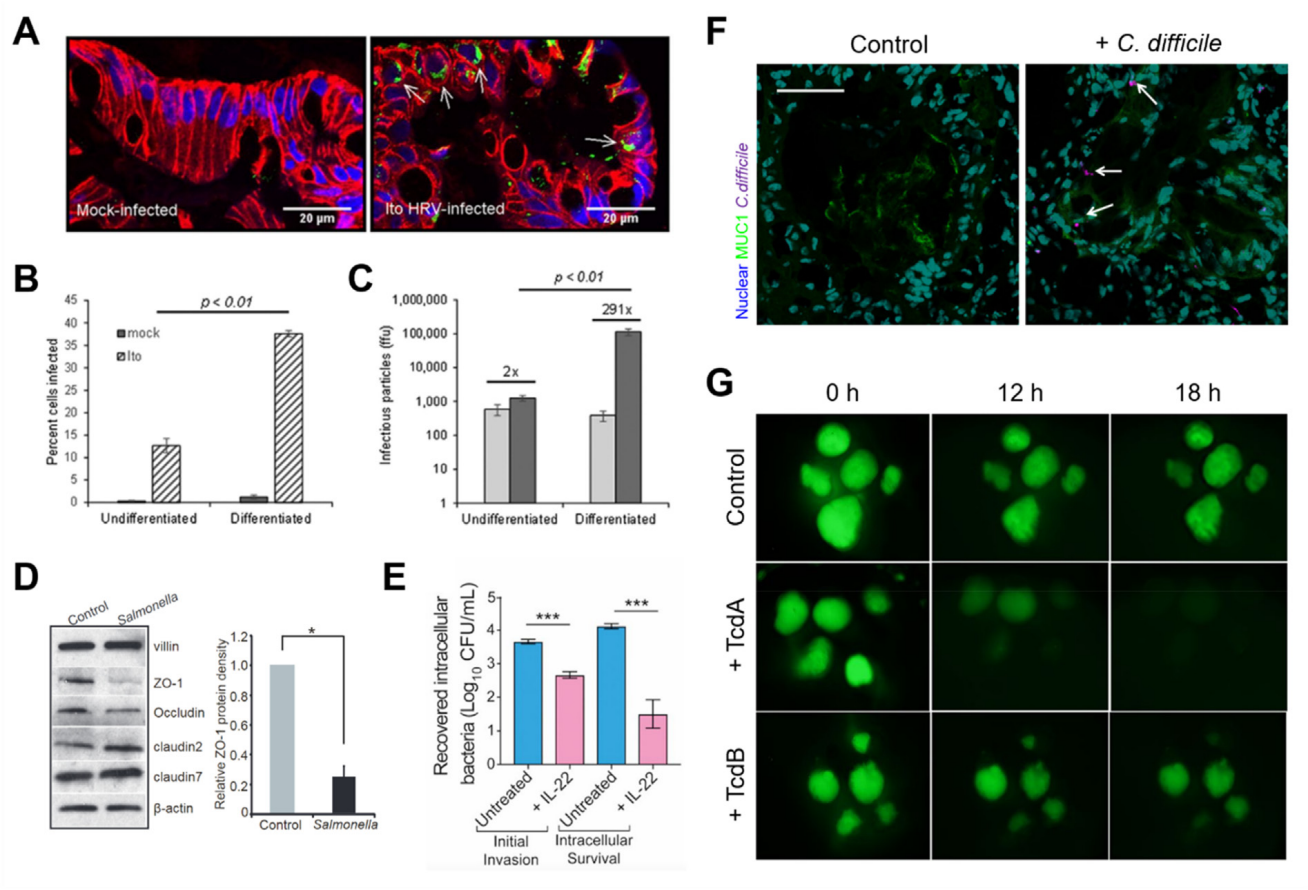
Pathogenic microbe-infected small intestinal organoids. (A) Immunofluorescence images of human rotavirus (HRV; strains Ito)-infected (right) organoids [60]. (B) The presence of intracellular rotavirus antigen in single cells from undifferentiated and differentiated organoids analyzed by flow cytometry [60]. (C) The amount of infectious virus in undifferentiated and differentiated organoids [60]. (D) Western blot analysis of tight junction proteins (Zo-1, Occludin, Claudin-2, and Claudin-7) in *Salmonella*-infected organoids [67]. (E) Gentamicin protection assays of IL-22-treated *Salmonella*-infected organoids [65]. (F) Confocal images of organoids injected with healthy stool and *C. difficile*. Scale bar, 50 μm [68]. (G) Representative images of fluorescein isothiocyanate (FITC, green)-dextran leakage from the lumen of organoids treated with *C. difficile* toxin tcdA and tcdB [23].

Viruses are infectious microorganisms that need to penetrate living host cells for replication and proliferation [58]. Co-cultures of organoids and viruses have emerged as *in vitro* models for studying the pathophysiology of viruses and development of therapeutics. Many studies have demonstrated that viruses can multiply within intestinal organoids and successfully infect intestinal cells (Fig. 3A) [19,[59], [60], [61]]. Currently, no available therapeutic modalities, such as specific treatment method, antibiotics/antiviral drug regimens, or intestinal motility inhibitors, exist to treat infections by rotavirus, the most common viral pathogen that causes diarrhea and enteritis, or norovirus, the virus that induces acute gastroenteritis. Up to 50% cells of differentiated intestinal organoids, which exhibit a “villi-like” epithelial phenotype, were infected with rotavirus, whereas a significantly lower proportion of undifferentiated “crypt-like” immature organoids were infected by the same pathogen (Fig. 3B and C) [60]. The administration of antiviral agents inhibits rotavirus in infected intestinal organoids, indicating that virus-infected organoids recapitulate the *in vivo* environment more closely than the cell line model [19]. Further, the necessity of bile for the replication of human norovirus in organoids and the enhancement of norovirus replication in the intestinal epithelium lacking an interferon response have been confirmed using genetically modified intestinal organoids [61]. Similarly, in a reovirus-infected intestinal organoid model, the genetic manipulation of type I and type III interferon receptors inhibited reovirus infection responsible for gastrointestinal disorders; and the antiviral activity induced by type III interferon was highly dependent on the mitogen-activated protein kinases (MAPK) signaling pathway, unlike type I interferon [62].

*Salmonella* is an enterobacterial pathogen that invades the intestinal mucosal surface, causing massive damage, along with gastrointestinal infections and typhoid outbreaks, resulting in thousands of deaths worldwide [63]. Microbial interactions of *Salmonella* with the gut have been analyzed using a microinjection into the lumen of intestinal organoids [22,[64], [65], [66]]. *Salmonella*-infected organoids exhibited inflammatory responses and the disruption of the epithelial tight junction *via* the expression of multiple genes and activation of the NF-κB pathway (Fig. 3D) [67]. The pretreatment of intestinal epithelial cells with IL-22 cell priming in patient-derived organoids carrying an IL10RB gene mutation that inactivates the IL-22 receptor inhibited early viral invasion and induced a barrier phenotype for more effective control of *Salmonella* infection (Fig. 3E) [65]. In addition, the activation of naturally secreted α-defensin in the complex environment of the intestinal lumen protected the organoid epithelium; gene expression profiling and biochemical assays showed that interleukin-22 (IL-22) induced the release of antimicrobial peptides and chemokines and aided in the maintenance of the defensive intestinal epithelial barrier in response to infection.

Intestinal organoids infected with pathogens have also been used to confirm that intestinal epithelial cells play a key role in regulating intestinal homeostasis and restraining pathogens. Patients infected with *C. difficile*, an enterobacterial pathogen, show a decrease in the expression of *MUC2*, a major indicator of a defect in the protective mucosal barrier against pathogen invasion. Intestinal organoids decrease the production of *MUC2* when infected with *C. difficile*, and the pathogen binds to *MUC1* to deliver the toxin (Fig. 3F) [68]. In addition, the cell line model showed only a higher toxicity of *C. difficile* toxin TcdB than another toxin TcdA, whereas organoids demonstrated that TcdA exhibited more potent toxicity than TcdB, causing a disruption of the intestinal epithelial barrier function (Fig. 3G) [23,69,70]. This result is consistent with those reported in previous studies on the inactivation of the GTPase Rap that regulates cell-cell junctions through TcdA [71].

**Fig. 4:**
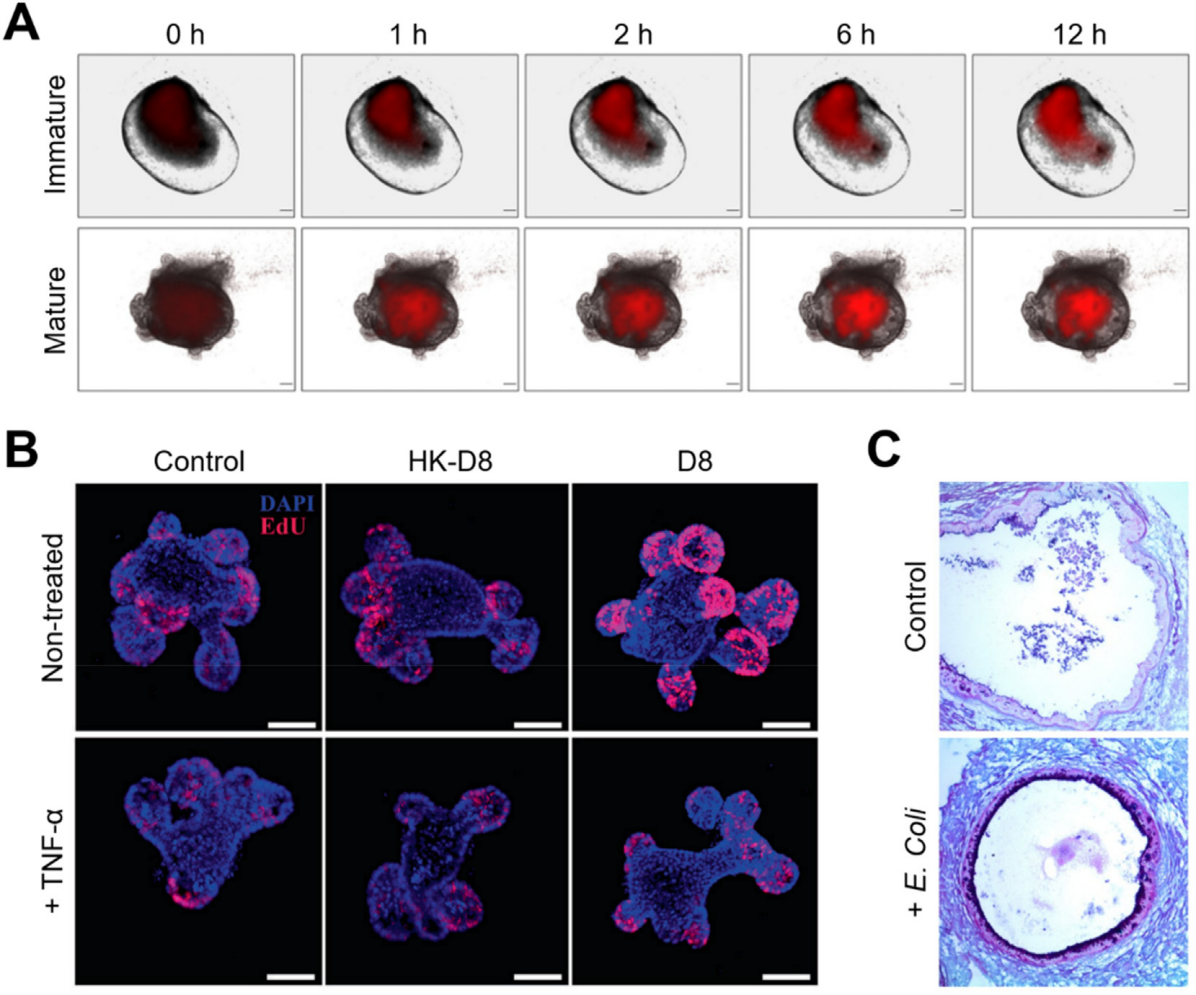
Symbiotic bacteria-co-cultured small intestinal organoids. (A) Time-lapse images of immature and mature organoids after microinjection with red fluorescence protein (RFP)-expressing *L. plantarum*. Scale bar, 200 μm [72]. (B) Immunofluorescence images of control (Ctrl), *L. reuteri* D8 (D8)-infected and heat killed D8 (HK-D8) organoids with (top) or without (bottom) the treatment of TNF-α [73]. (C) Histological images of periodic acid-Schiff (PAS)- and Alcian blue-stained organoids microinjected with *E. coli* [74].

*Lactobacilli*, probiotic symbiotic bacterial species residing in the human small intestine, have been extensively used to maintain the integrity of the intestinal epithelial barrier and treat a variety of intestinal disorders. Despite their ability to exert health-promoting effects and beneficially modulate gut immunity, the limited access to human small intestine has made it difficult to establish research models for their study. Recently, a co-culture system of intestinal organoids and symbiotic bacteria *Lactobacillus reuteri* and *Lactobacillus plantarum* has been developed to demonstrate higher proliferation and viability when *Lactobacilli* were microinjected into the lumen of mature intestinal organoids rather than immature organoids (Fig. 4A) [72]. In addition, a co-culture of *Lactobacilli*-infected intestinal organoids with intestinal lymphocytes has shown that the colonization of lactobacilli promotes the proliferation of intestinal epithelium and repairs damaged intestinal mucosa by increasing the number of Lgr5^+^ and lysozyme^+^ cells (Fig. 4B) [73]. It also stimulates the secretion of IL-22 from the lymphocytes eventually inducing the phosphorylation of the signal transducer and activator of transcription 3 (STAT3) protein (Fig. 4C) [74]. The co-culture of *Lactobacillus rhanmosus* in small intestine organoids led to increase the degree of Paneth cell differentiation and the expression level of Ki-67, indicating the potential of the application of microorganisms to enhance the growth and differentiation of intestinal stem cells [75]. To study how *Lactobacillus* affects infection of *Salmonella*, which has shown to cause villi annihilation and collapse of intestinal organoids [22,[64], [65], [66], [67]], the small intestine organoids were infected with Salmonella after pre-treatment with *Lactobacillus acidophilus* [76]. *L. acidophilus* inhibited *Salmonella* invasion, as well as showed the ability to protect the intestinal mucosa from pathogen infection. *L. acidophilus* pre-treatment increased the expression of MUC2 to protect the invasion of Salmonella, and reduced the damage of intestinal organoids by tumor necrosis factor-α and the ratio of apoptotic cells, thereby positively affecting the formation of intestinal organoids. In addition, *L. acidophilus* suppressed the excessive proliferation of goblet cells and Paneth cells induced by *Salmonella*, and ameliorated overactivation of the Wnt/β-catenin pathway upon contact with toll-like receptor 2. Similarly, another organoid co-culture model confirmed that *L. reuteri* D8 increase the mRNA expression of R-spondin, Wnt3 and Lrp5 to activate the Wnt/β-catenin pathway and to increase Lgr5 protein levels and Lgr^+^ cell numbers [77]. The surface area of intestinal organoids was significantly increased in *L. reuteri* D8 co-culture, while stimulating intestinal epithelial proliferation through the increases in expression of c-Myc, cyclin and Ki-67. Also, the recovery function of *L. reuteri* D8 was confirmed in pathological conditions such as intestinal organoid damage and destruction induced by tumor necrosis factor (TNF), where *L. reuteri* D8 improved the expression of Lgr5, Olfm4, and Ascl2 which were inhibited by TNF by decreasing TNF expression. These results verified that commensal bacteria are effective in restoring intestinal damage and maintaining homeostasis after infection and pathological damage caused by exogenous bacteria, suggesting potential as a promising therapeutic technique in the future. In another study, co-culture with *E. coli*, a non-pathogenic commensal bacteria common in the intestine, prevented the loss of E-cadherin expression in intestinal organoids, increased production of reactive oxygen species, and promoted apoptosis [78]. The intestinal organoid model has also been shown to be a comparative analytical model for non-pathogenic and pathogenic microbes; the colonization of intestinal organoids with non-pathogenic *E. coli* activates antimicrobial defense pathways, including mucus production and genes related to toll-like receptor, cytokine, and NF-κB signaling. It also leads to an upregulated the genes associated with intestinal maturation and a loss of intestinal epithelial barrier function [24,74,79]. Taken together, the co-culture of intestinal organoids with various bacteria and viruses can serve as a useful tool that aids in understanding host-microbe interactions.

*Toxoplasma gondii* and *Giardia duodenalis* are the two most common parasites associated with protozoan diseases in humans and animals [[80], [81], [82], [83]]. Both parasites are zoonotic pathogens that mainly infect to the hosts through the small intestine. However, the interactions between parasites and small intestinal tissues are still elusive, and current approaches for studying the host-pathogen interactions depend mainly on the immortalized cell line-based 2D models [80]. To understand the mechanism of parasite infection, single and simultaneous infection of *T. gondii* and *G.* *duodenalis* was performed on organoid-derived monolayers (ODMs) [84]. From the measurement of transepithelial-electric resistance to confirm the disturbance of intestinal barrier function, no significant effect on barrier function was observed in ODM infected with *T. gondii*, whereas *G. duodenalis*-infected ODM exhibited a loss of barrier function through a decrease in epithelial resistance. In addition, unlike *T. gondii* infection that shows no clear change in the expression of tight junction proteins such as zonula occluden 1 (ZO-1) and occludin, there was significant changes in tight junction proteins in *G. duodenalis* infection and co-infection of *T. gondii* and *G. duodenalis*.

#### 3.1.2 Colon

**Fig. 5:**
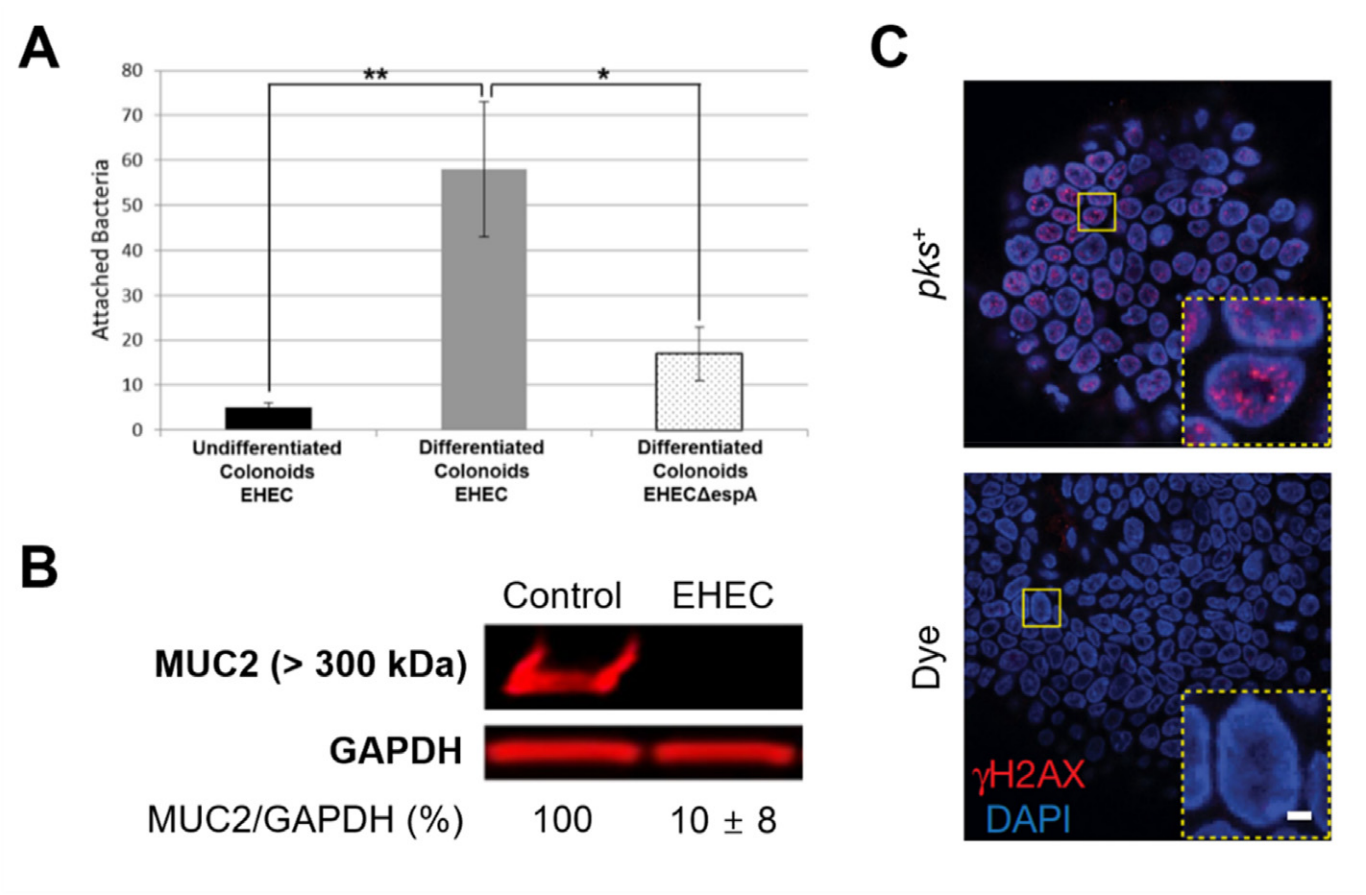
*E. coli*-infected colon organoids. (A) Quantification of enterohemorrhagic *E. coli* (EHEC) or EHEC*ΔespA* bacteria associated with organoids [43]. (B) MUC2 expression in EHEC-infected organoids [43]. (C) Immunofluorescence images for DNA damage induction (γH2AX) of *pks*^+^*E. coli*-co-cultured colon organoids [85].

Enterohemorrhagic *E. coli* (EHEC) is a flesh-eating pathogen that causes various complications, such as hemorrhagic colitis and hemolytic uremic syndrome [6]. Few studies exist on the interaction between the colon and EHEC before the onset of complications, due to a lack of appropriate animal models and the presence of asymptomatic conditions upon immediate EHEC infection. Human colon organoids co-cultured with EHEC can therefore be a sophisticated pathophysiological model that enables molecular-level studies, facilitating the analysis of colon disease during the early phase (0–8 h) of EHEC infection. The differentiated organoids demonstrated a higher degree of EHEC colonization than their undifferentiated counterparts (Fig. 5A) [43]. The EHEC-infected organoids revealed that the thickness of the MUC2 mucus layer, a protective barrier between commensal bacteria and colonic epithelium, was rapidly reduced (< 6 h) after infection (Fig. 5B). Another study confirmed that DNA damage was induced *via* co-culture with *pks*^+^ *E. coli* that produces colibactin, a genetic toxin (Fig. 5C) [85].

*Bifidobacterium adolescentis* is an anaerobic bacterium that colonizes the intestinal mucus layer and provides many health benefits to the host in a symbiotic-like relationship such as mucus production [86]. *B. adolescentis* was co-cultured on a monolayer derived from colon organoids to determine how *B. adolescentis* supports colon homeostasis and function through interaction with the colon [48]. *B. adolescentis* upregulated the expressions of MUC2 in colon epithelium and stem cell marker genes such as LGR5, AXIN2, and PTK7, which demonstrates the stem cell-promoting effects of *B. adolescentis*. In addition, successful colony formation was also observed thorough co-cultures with obligate anaerobic bacteria such as *Bactericides fragilis* and *Clostridium butyricum*. In particular, the co-culture with *Akkermansia muciniphila* revealed that *A. muciniphila* grew by decomposing the mucin layer without adding exogeneous mucin, thereby demonstrating the biological effect of epithelium on intestinal bacteria.

One of the most widely used probiotic strains, *Lactobacillus rhamnosus* GG (LGG), has been known to improve symptoms of irritable bowel syndrome (IBS); however, the mechanism has not been clearly elucidated [87,88]. The LGG-co-cultured-organoid model analyzed how LGG affects the intestinal epithelial barrier lost by interferon-γ, a major cytokine in IBS that disrupts intestinal barrier homeostasis [89]. Upon injection of interferon-γ into the organoids without LGG infection, the function of intestinal epithelial barrier was disrupted, resulting in decreased gene expression of tight junction ZO-1 and occludin, and rapid loss of barrier integrity. However, in LGG-co-cultured organoids, the expression of ZO-1 and occluding was shown similar to that of normal organoids, while exhibiting normalization of the epithelial barrier function.

#### 3.1.3 Liver

**Fig. 6:**
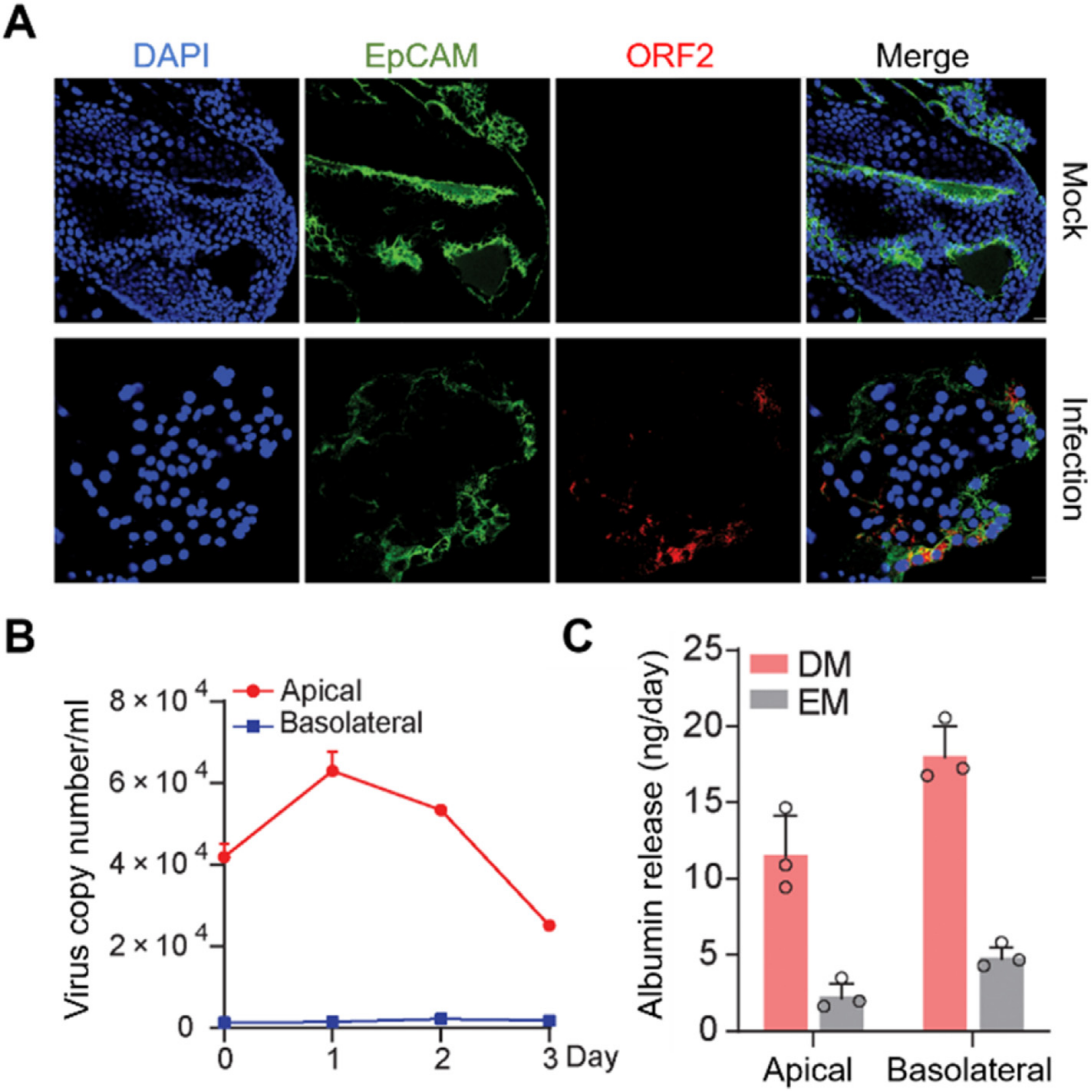
HEV-infected liver organoids. (A) Immunofluorescence staining for HEV ORF2 protein in intrahepatic cholangiocyte organoids on day 3 after infectious HEV particles [92]. (B) Quantification of released viruses from polarized cells inoculated with HEV particles. Scale bar, 20 μM [92]. (C) Release of albumin into apical and basolateral compartments from polarized cells cultured in expansion medium (EM) with cholangiocyte phenotype or differentiation medium (DM) with hepatocyte phenotype (n = 3) [92].

Hepatotropic viruses are representative microorganisms that pose significant public health problems with a great impact on global mortality, and thus the models to understand the host-virus interactions and the mechanisms of viral pathogenicity are urgently needed [90]. Among these hepatotropic viruses, hepatitis E virus (HEV) is one of the most common causes of acute hepatitis and acute liver failure [91]. To understand the mechanism of HEV, the intrahepatic cholangiocyte organoids derived from the intrahepatic biliary compartment were co-cultured with the HEVs (Fig. 6A) [92]. HEV has been known to be replicated from hepatocytes and transported across the apical membrane into the bile ducts; in the HEV-infected organoid model, hepatocyte-secreted albumin is directed basolaterally, whereas HEV particles were mostly emitted toward the apex (Fig. 6B and C). The intrahepatic cholangiocyte organoids showed anti-HEV effects in response to the treatment of ribavirin and mycophenolic acid. HEV replication stimulated robust type I interferon responses, indicating the potential for interferon α-based HEV suppression therapy.

Similar to HEV, hepatitis B virus (HBV) is a type of hepatotropic virus and can cause persistent and chronic infections in humans [93]. The liver organoids infected with HBV showed a decrease in membrane microvilli, a hallmark of fibrosing liver disease, and upregulation of epithelial mesenchymal transition markers, leading to organoid hepatic dysfunction [94]. In another study, HBV-infected liver organoids exhibited an upregulation of mRNA expression of the organoid-derived signature genes, and differentiated organoids showed higher HBV infection efficiency than immature organoids in expansion medium due to overexpression of sodium taurocholate co-transporting polypeptide [95]. The HBV infection early gene signatures and biomarkers could be identified through comparative transcriptome analysis of healthy organoids and HBV-infected patients-derived organoids. In particular, by identifying the gene signatures of hepatocellular carcinoma and early liver cancer, the organoid infection model could be utilized as a promising model to predict development of the carcinoma and to understand the molecular mechanisms underlying HBV replication in primary cells.

#### 3.1.4 Stomach

**Fig. 7:**
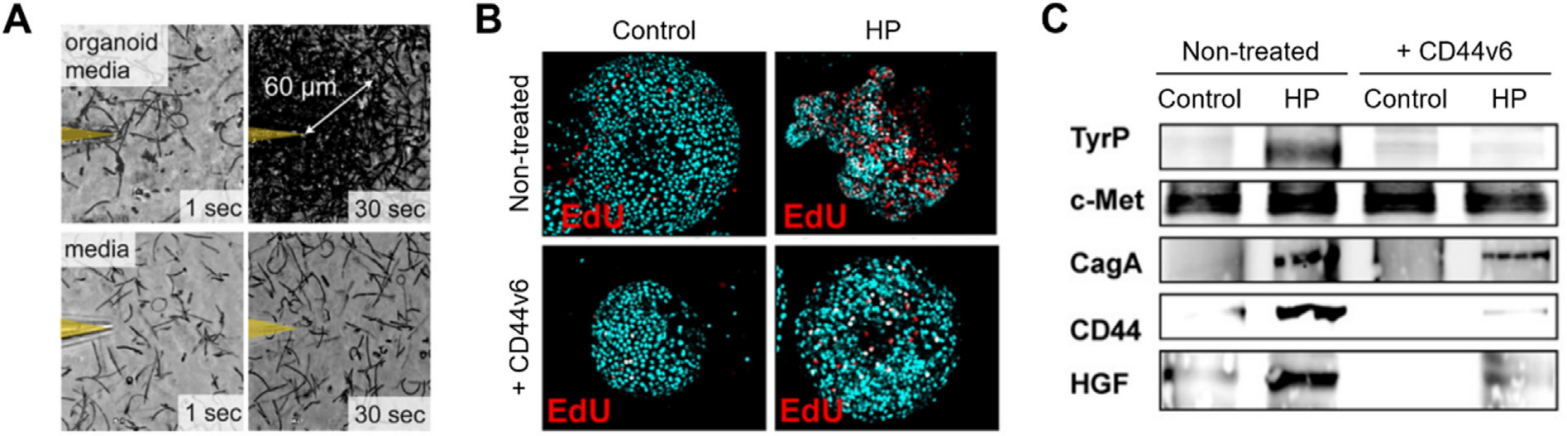
*H. pylori*-infected gastric organoids. (A) Motility tracings of *H. pylori*'s response to organoid-conditioned media (top) and non-conditioned media (bottom) at 1 s and 30 s post-injection [102]. (B) Immunofluorescence images and (C) western blot analysis of EdU^+^ cells in control (CON), *H*. *pylori* (HP)-infected, CD44v6 neutralizing antibody-treated (CD44v6), and CD44v6 neutralizing antibody-treated HP-infected (CD44v6+HP) organoids [100].

*Helicobacter pylori*, a microaerobic spiral-shaped bacterium found in the human stomach, can cause gastric adenocarcinoma and various stomach disorders [96,97]. *In vitro* studies of *H. pylori* have mainly relied on the use of transformed cell lines as infection models; however, this approach is particularly inadequate for understanding the mechanisms of cancer development due to their artificial physiological features. When *H. pylori* is microinjected into the lumen of gastric organoids to study its interactions with the gastric epithelium [[98], [99], [100], [101]], metabolites released from gastric organoids by a bacterial chemoreceptor (TlpB) rapidly attract bacterial cells (Fig. 7A) [102]. In addition, the co-culture of organoids with *H. pylori* lacking the virulence factor CagA has been employed to confirm that CagA forms a complex with the C-Met receptor after translocation to gastric epithelial cells, disrupting the epithelial layer [99,103]. In another study, the suppression of the differentiation cluster gene CD44 in *H. pylori*-infected gastric organoids led to a decrease in *H. pylori*-induced hyperproliferation and cancer induction (Fig. 7B and C) [100]. NF-κB activation led to the *H. pylori* infection-induced expression of sonic hedgehog (Shh) [101]. Collectively, the gastric organoid model can be used to elucidate potential mechanisms of *H. pylori* infection as well as to study various gastric pathologies.

#### 3.1.5 Gallbladder

**Fig. 8:**
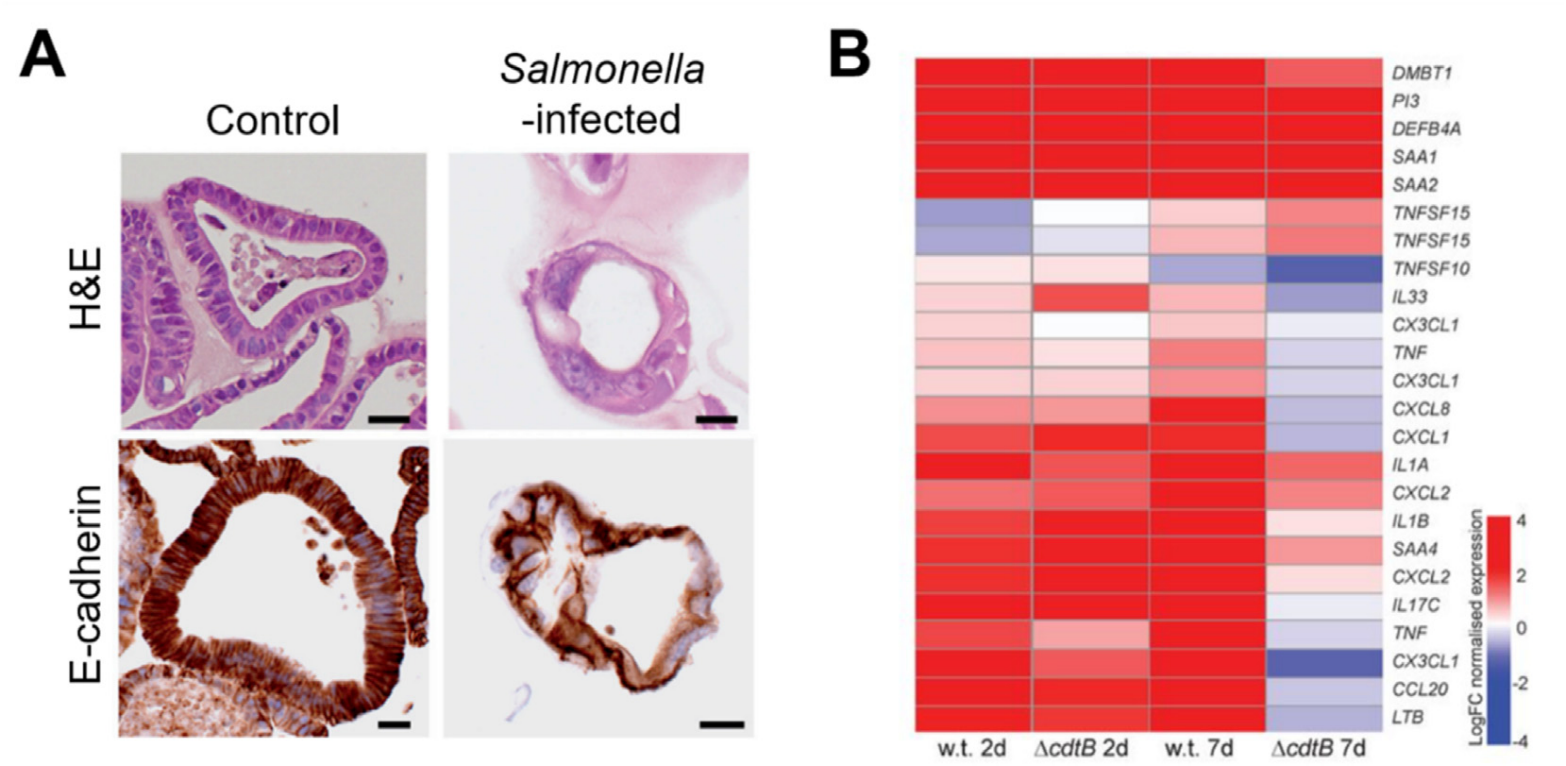
*Salmonella*-infected gallbladder organoids. (A) Histological images of H&E (top)- and E-cadherin (bottom)-stained gallbladder organoids. Scale bar, 20 μm [35]. (B) Heat map for manually selected NF-κB target genes, with a comparison of wild type (w.t.) *Salmonella* and *SalmonellaΔcdtB* infections at 2 and 7 days post-infection [105].

*Salmonella* infection has been reported as a main risk factor for gallbladder carcinoma; however, the direct association and mechanism are not precisely known yet [104]. Infected gallbladder organoids show a loss of cohesion and polarity and irregular nuclear morphology [35]. Meanwhile, the overexpression of c-MYC and inactivation of the Arf/TP53 pathway induce the transformation of *Salmonella*-infected cell. Moreover, *Salmonella* promotes cell division and phenotypic transition of infected cells by activating the MAPK and protein kinase B (AKT) pathways (Fig. 8A). In addition, the prolonged maintenance of gallbladder organoid culture was shown to be dependent on R-spondin that mediates activation of the Wnt/β-catenin signaling pathway and regeneration of gallbladder epithelium by Lgr5^+^ cells [105]. In addition, the role of *Salmonella* toxin ΔcdtB that induces typhoid was demonstrated through NF-κB target gene analyses. These gallbladder organoid-based studies have opened new avenues to understand the direct relationship of bacteria with oncogenesis (Fig. 8B).

### 3.2 Respiratory system

Lung is the main route of *Mycobacteria*, and its infection causes severe morbidity and mortality. *Mycobacterium tuberculosis*, the main cause of tuberculosis, has been extensively shown to be associated with diseases such as pulmonary complications and autoimmune diseases [106]. Meanwhile, *Mycobacterium abscessus*, does not cause tuberculosis but can cause immunosuppression and a wide range of diseases [107]. The airway organoids infected with *M. tuberculosis* and *M. abscessus* were constructed *via* microinjection [108]. Upon mycobacteria infection, downregulation of NF-Κb regulatory genes led to low expression of mucins such as MUC5B and MUC4. In addition, co-culture of mycobacteria-infected organoids with macrophages showed that macrophages migrated to the basal edge of the organoid to capture and ingest bacteria, thus confirming that macrophages are essential for tuberculosis diseases.

Human parainfluenza virus type 3 (HPIV3) infects lower respiratory tract epithelial cells and causes childhood bronchiolitis and pneumonia, however, its effect on the lung has not been precisely known [109]. Most current studies on HPIV3 rely on immortalized cell lines; a realistic lung-like model is necessary to understand the interaction of the lung with vial surface glycoproteins that mediate the entry of viruses into host cells [109,110]. HPIV infection was performed either by depositing an inoculum under Matrigel adjacent to the lung organoid tissue or by microinjection, both of which confirmed that HPIV3 targeted the alveoli [111]. The infected cells exhibited viral entry without syncytium formation, and particles released by the virus diffused to adjacent locations within the alveolar structure.

Respiratory syncytial virus (RSV) is a virus that causes acute lower respiratory tract infection in infants; despite extensive research efforts, there is no licensed vaccine or effective antiviral agent yet, and little is known about the immunobiology of RSV infection [112]. In the human lung organoids, RSV infection was confirmed in smooth muscle actin-positive and vimentin-positive expressing cells, but not in tubulin-positive expressing cells [113]. Similar to cilia formation known as a cytopathic effect of the virus in mature airways, RSV infection reduced the population of FoxJ1-positive ciliated cells and altered organoid structures such as E-cadherin destruction. In addition, by comparing RSV-infected and non-infected organoids, high expression of proteins belonging to the category of neutrophil degranulation, which influence the pathogenesis of RSV-induced inflammation, was confirmed. The RSV-infected cells have shown to swell and flow into the lumen of organoid branching structure, which is similar to archival pathology [114,115].

Influenza A virus is a pathogenic virus that can infect a variety of hosts, such as birds and mammals, including humans [116]. However, the model to predict cross-species virus transmission potential and influenza infectivity remains still lacking. The human airway organoids model replicates the avian influenza H7N6/Ah virus with high human infectivity more strongly than the avian influenza H7N2 virus with low human infectivity, confirming the possibility of distinguished cross-species virus transmission [117]. A comparative analysis of H1N1pdm and H1N1sw in the airway monolayer derived from organoids also revealed a very high virus transmission potential. Even though the viral titer of the basolateral medium was relatively low due to the epithelial barrier formed in the 2D monolayer and the preferential release of virus from the apical side of cells, the human airway organoids demonstrated differences in the replication capacity between viruses on both the apical and basal sides.

**Fig. 9:**
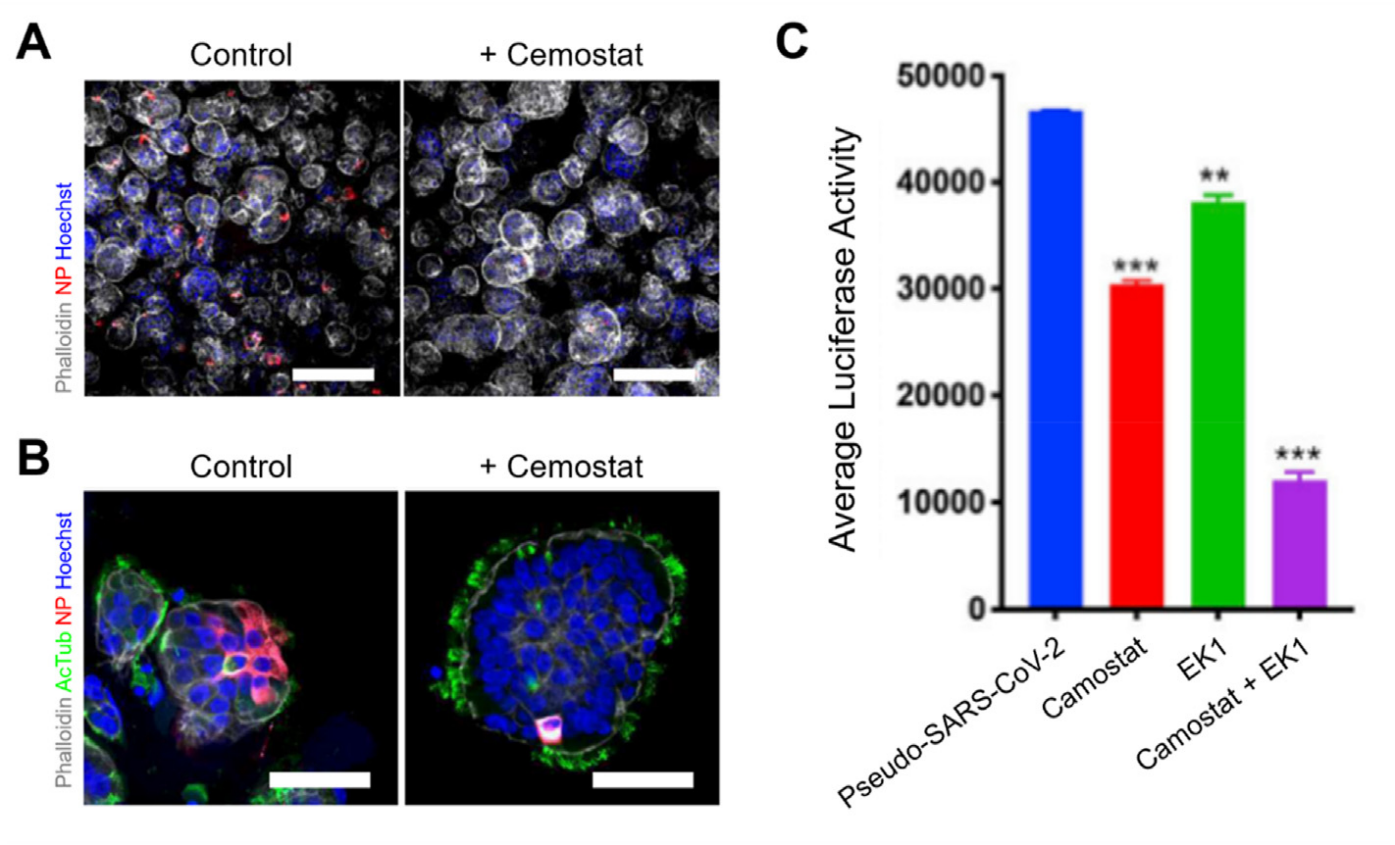
SARS-CoV-2-infected lung organoids. Immunofluorescence images of (A) bronchiolar and (B) bronchial organoids pretreated with Camostat [120]. (C) Inhibition of SARS-CoV-2 infection by treatment with viral entry inhibitors [118].

COVID-19 is a contagious respiratory disease caused by infection with a novel coronavirus, severe acute respiratory syndrome coronavirus 2 (SARS-CoV-2). It can lead to fatal disorders, including severe pneumonia [5]. To overcome the COVID-19 pandemic, a robust and practical *in vitro* model system for SARS-CoV-2 virus etiology is necessary. Since SARS-CoV-2 mainly infects the respiratory tract, lung organoids have been most extensively developed in recent years [[118], [119], [120], [121], [122]]. SARS-CoV-2-infected lung organoids demonstrated that the expression of serine protease 2 (TMPRSS2) is essential for priming the spike (S) protein of angiotensin converting enzyme-2 (ACE2) of coronavirus. The entry and replication of coronaviruses are effectively inhibited through pretreatment with Camostat, a serine protease inhibitor, confirming TMPRSS2 as the main entry route *in vivo* (Fig. 9A and B) [120]. In addition, the high-throughput screening of lung organoids has enabled the identification of drugs inhibiting the entry of various coronaviruses (Fig. 9C) [118]. In addition to the respiratory system, studies on organoid-based coronavirus infections have been conducted for various organs, including the stomach [45], small intestine [123,124], and cerebrum [118], to develop models for understanding the pathogenesis of COVID-19 and testing new therapeutic drugs.

*Aspergillus fumigatus* is the most important pathogenic fungus in humans causing various lung diseases including allergic bronchopulmonary aspergillosis, chronic pulmonary aspergillosis and invasive aspergillosis [125]. The infection of *A. fumigatus* led to upregulation of stimulatory inflammatory cytokine expression through pathogen recognition receptor in lung organoids, with expressions of tissue inflammation markers such as cyclooxygenase, matrix metalloproteinase 9, and the acute-phase protein C-reactive proteins [126]. These results suggest that the potential of microbe-co-cultured organoids models to mimic the immune function and immunocompetent tissue microenvironment of organs.

### 3.3 Nervous system

**Fig. 10:**
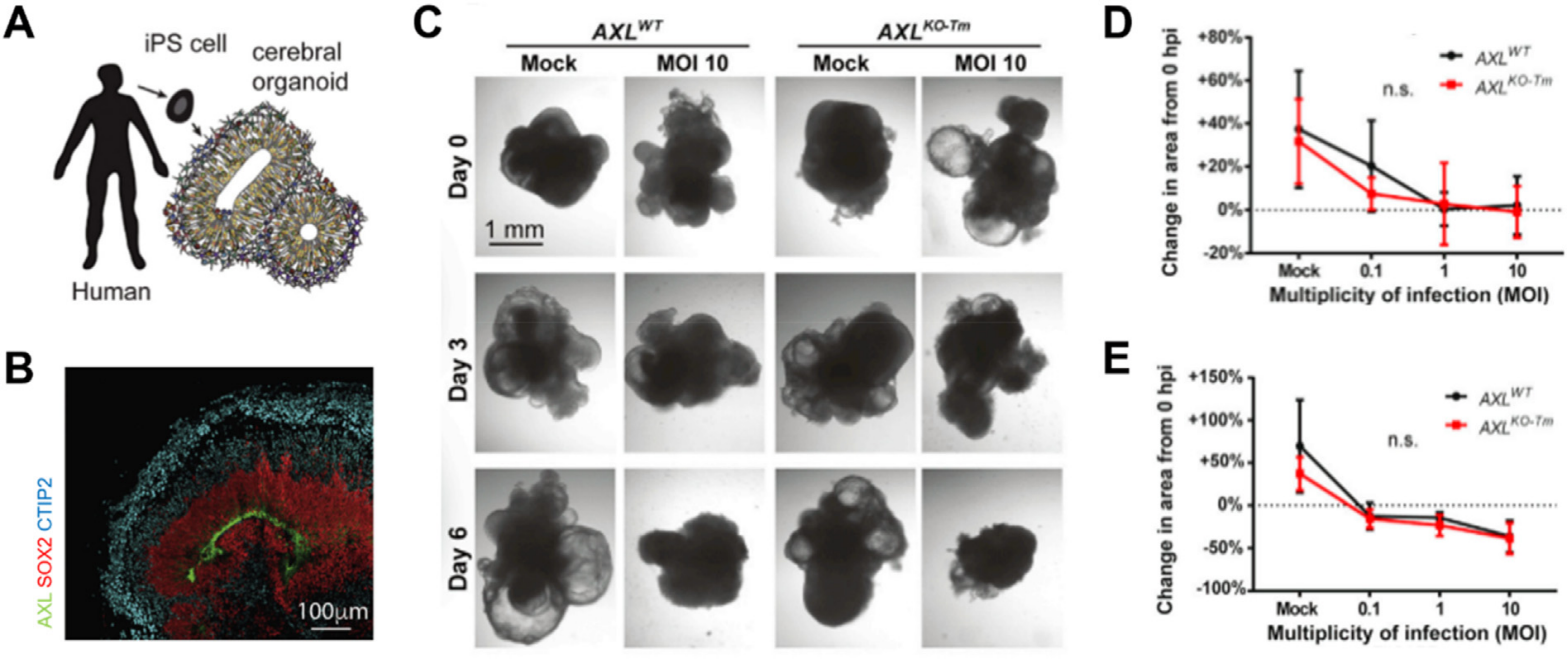
Zika virus-infected brain organoids. (A) Schematic illustration for generation of induced pluripotent stem cell-based cerebral organoids [130]. (B) Immunohistochemistry image of AXL expression in the lumen of organoids after 70 weeks of culture [130]. (C) Bright-field image of ZIKA-infected organoids for wild type AXL (AXL^WT^) and transmembrane domain knocked-out AXL (AXL^KO−Tm^) [131]. Quantified size of cross-sectional organoids at (D) 72 h and (E) 144 h post-infection [131].

Zika virus, a mosquito-borne flavivirus, causes birth defects including microcephaly and is associated with meningitis and myelitis [34]. Zika virus has been demonstrated to preferentially target neural progenitor cells (NPCs) across the placenta [127,128], leading to cell death *via* apoptosis or autophagy by inducing the depletion of progenitors due to premature differentiation of NPCs causing centromere perturbation. Microcephaly results from ventricular region destruction, cortical damage, and neurogenesis disorders [34,128,129]. Though the AXL receptor has been increasingly considered as a major attachment factor for the entry of Zika virus and NPC death, the role of AXL still remains elusive. To understand the effect of AXL on Zika virus pathogenesis, a cerebral organoid model capable of expressing AXL has been developed (Fig. 10A and B) [130]. Nevertheless, another study described that AXL is not essential for Zika virus infection in NPCs and organoids with genetically excised AXL (Fig. 10C–E) [131]. As these results indicate, the inhibition of AXL alone does not seem to be enough to ameliorate Zika virus infection during brain tissue development. In addition, the inhibition of the innate immune receptor toll-like receptor 3 reduced the phenotypic effects of Zika virus infection [132]. Though preclinical studies on Zika virus have not precisely identified drugs to treat or prevent the infection [133,134], a screening using approximately 6000 compounds on brain organoid models has identified the caspase-3 activity inhibitors, Emricasan and Niclosamide, to be effective in inhibiting the death of NPCs and proliferation of Zika virus [25]. Besides, a multi-well rotating bioreactor SpinΩ was developed for the easy and cost-effective establishment of a Zika-infected organoid model that allows brain region-specific analysis [135]. In addition to Zika virus, pathogenic mechanisms of various microbes, such as human immunodeficiency virus and herpes simplex virus, have been studied using brain organoids [136,137].

Toxoplasmosis is a parasitic disease that results from *T. gondii* infection; upon infection with *T. gondii*, it can penetrate to circulating cells such as macrophages and cross the blood-brain-barrier [81,82]. It has been reported that humans infected with *T. gondi*i develop abnormal neurocognitive behaviors and mental disorders. However, no drug has been approved for Toxoplasmosis yet, and the study of *T. gondii* infection in 2D models showed non-physiological pressures and tensions that can alter processes of *Toxoplasma* infection [138]. To overcome these limitations, *T. gondii*-infected cerebral organoids model was developed; *T. gondii* formed a cyst-like structure within the organoid, and tachyzolite was differentiated into bradyzolite; thereby proving that it summarizes the life cycle stages of the parasite [139]. In addition, in the cerebral organoids, differentiated neuronal cells exhibited a higher priority than radial glial cells for the infection of *T. gondii*.

### 3.4 Reproductive system

**Fig. 11:**
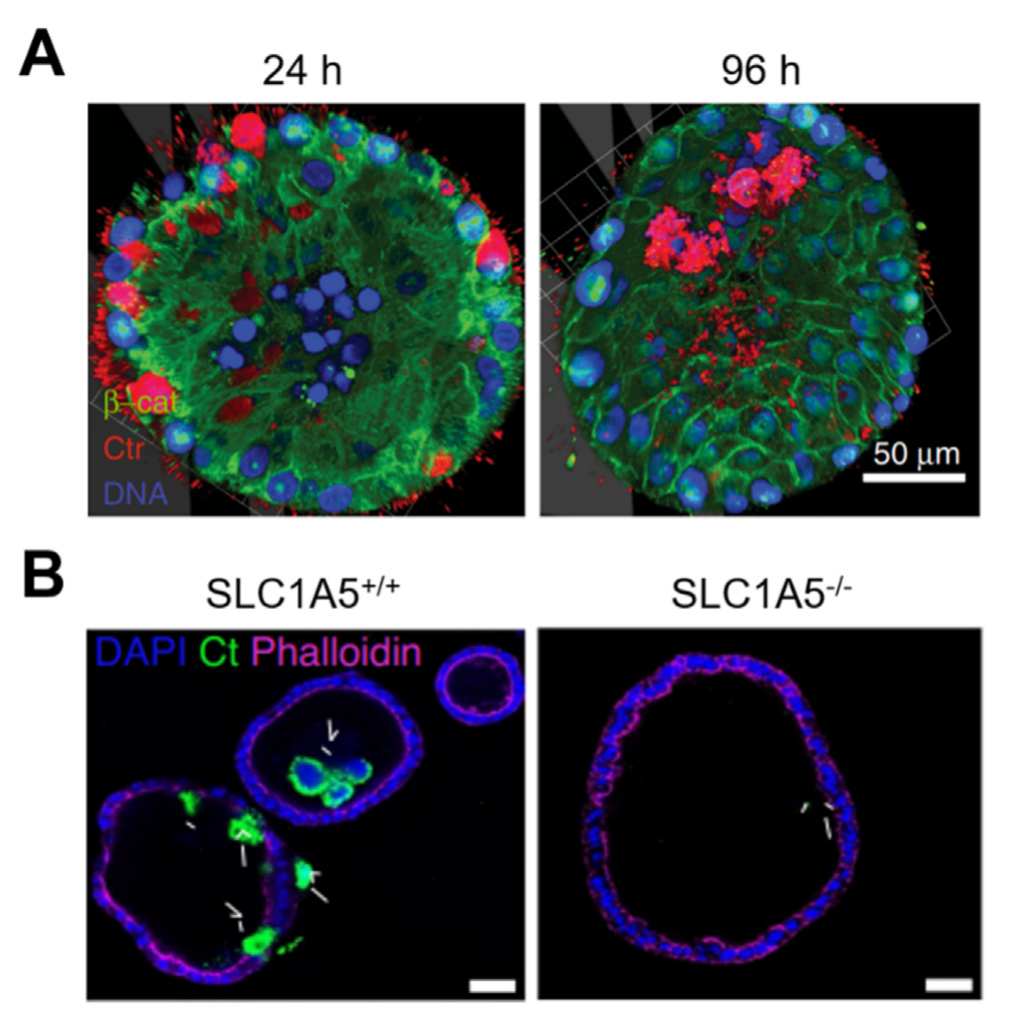
*C. trachomatis*-infected fallopian tube organoids. (A) Whole-mount 3D images for the localization of bacteria in acute *C. trachomatis*-infected organoids. (*C. trachomatis*, red; β-catenin, green; nuclei, blue). Scale bar, 50 μm [140]. (B) Immunofluorescence images of *C. trachomatis*-infected organoids derived from SLC1A5^+/+^ and SLC1A5^−/−^ mice [145].

*Chlamydia trachomatis* not only induces sexually transmitted diseases but also causes chronic infections, fallopian tube scarring, and possible infertility [140,141]. *C. trachomatis* has been shown to disrupt host cell metabolism and cause DNA damage *in vitro* [142,143]. Several studies have reported structural damage to the *C. trachomatis*-infection fallopian tubes but the mechanism still remains unclear [144]. The *C. trachomatis*-infected fallopian tube organoids models have been established to study the pathological relationship of *C. trachomatis* and salpingitis [140,145]. In this model, *C. trachomatis* was mainly found in the lumen of organoids, and the infection induced a change in the cell composition of the organoid monolayer (Fig. 11A) [140]. In addition, the adsorption of glutamine is critical for the synthesis of peptidoglycans that play important roles in the proliferation of *C. trachomatis*; their proliferation was inhibited in fallopian tube organoids, in which SLC1A5, a glutamine transporter, was knocked out (Fig. 11B) [145].

Human papillomavirus (HPV) is one of the major causes of cervical carcinogenesis [[146], [147], [148]]. Upon HPV infection, co-factors like immune status, hormones, and co-infections are known to influence the development of cervical cancer. To understand the interaction between HPV and bacterial pathogen *C. trachomatis*, a co-infection model was developed *via* infection of *C. trachomatis* to the ectocervix organoid induced by integration of E6E7 oncogene into the host genome [149]. The HPV E6E7 inhibited the redifferentiation of reticulate bodies into elementary bodies, as well as interfered with *C. trachomatis* development and reduced persistence. The *C. trachomatis* suppressed mismatch repair gene expression by proteasomal degradation of transcription factor E2F1. These results suggest that the co-existence of HPV and *C. trachomatis* in stem cells can promote cervical carcinogenesis and tumor progression. Nevertheless, direct infection of HPV into organoids, not through direct method such as genetic manipulation, has progressed only with oral mucosal organoids [150]; no studies have been performed on reproductive system organoids. Taken together, these approaches could be potentially useful to elucidate the pathogenesis of various diseases of the reproductive system, including serous ovarian cancer, the long-term effects of Chlamydial infection, and co-infections with pathogenic microbes.

### 3.5 Urinary system

**Fig. 12:**
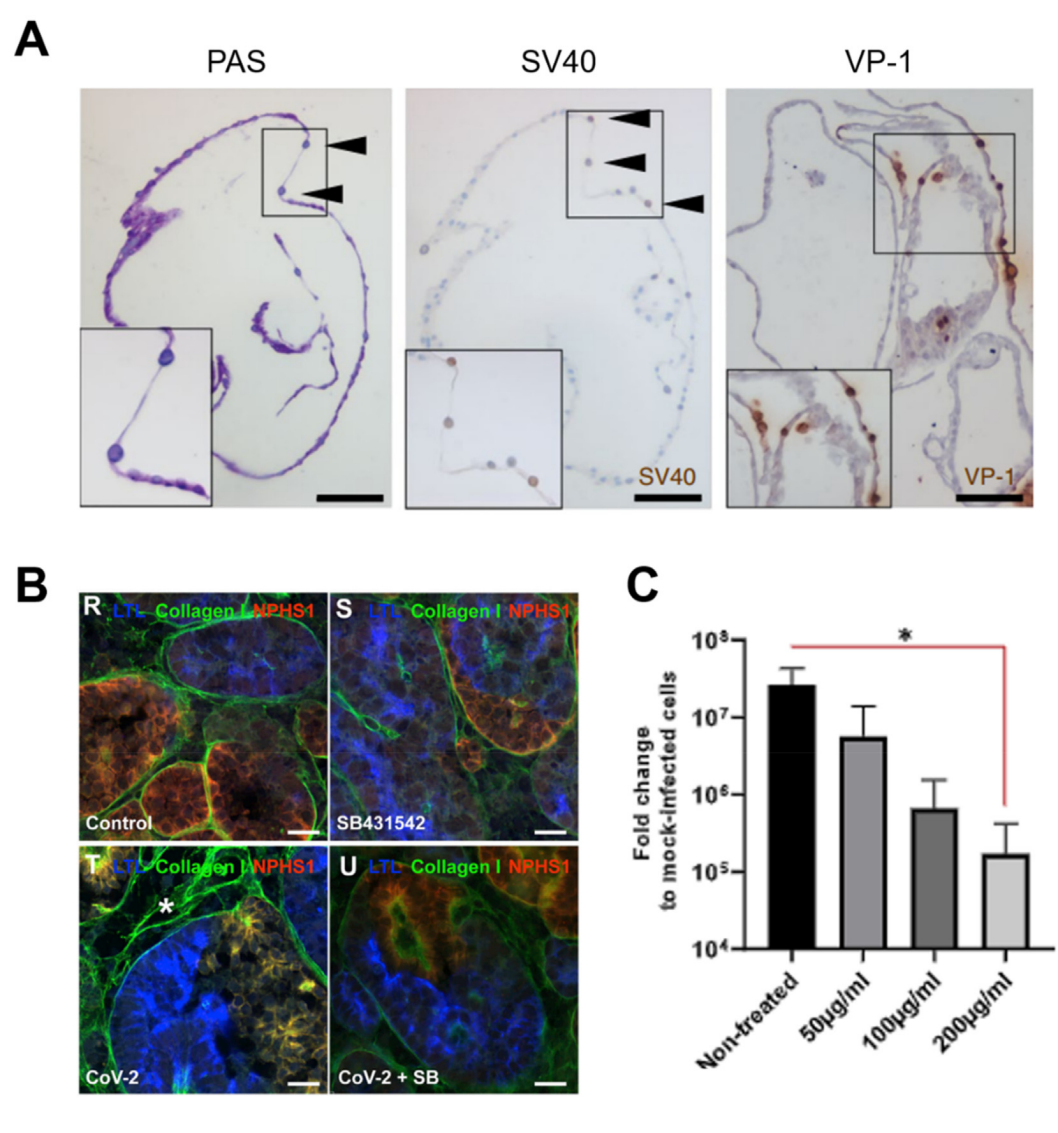
Virus-infected kidney organoids. (A) Histological images of periodic acid-Schiff (PAS)-, SV40 T antigen (early gene of BK virus)- and VP-1 (late gene of BK virus)-stained BK virus-infected tubuloids at 10 days post-infection. Black arrows indicate larger blue nuclei (intracellular basophilic viral inclusions) [152]. (B) Kidney organoids without any treatment (R, mock-infected) or with treatment using SB431542 (S), SARS-CoV-2 (T) and SARS-CoV-2 + SB431542 (U). Scale bar, 20 μm [154]. (C) Effect of human recombinant soluble ACE2 (hrsACE2) on SARS-CoV-2-infected kidney organoids [155].

Infection with BK virus, a tubule-specific virus, has no specific treatment and is one of main causes of nephropathy and disability in kidney transplant recipients [151]. Renal organoids infected with BK virus demonstrated an infection pattern similar to that of nephrotic tissue, and the nuclei of infected tubular cells double in size due to intranuclear basophilic viral inclusions [152]. In addition, the expression of an early gene of the BK virus, SV40/large T antigen, and a late gene VP-1 were detected in the BK virus-infected renal organoids (Fig. 12A).

SARS-CoV-2 infects and damages the cells of the kidney, an important target organ for COVID-19, but the mechanism remains unclear [153]. Coronavirus-infected renal organoids models have been constructed to identify the pathophysiological relevance; coronaviruses have been reported to infect renal tubular epithelial cells and induce acute injury and fibrotic remodeling, leading to renal failure and chronic kidney disease [154]. The SARS-CoV-2-infected renal organoids exhibited an upregulation in the expression of fibrosis-inducing collagen type I, and this upregulation could be reduced by blocking TGF-β receptor I (Fig. 12B). Further, the administration of human recombinant soluble ACE2 (hrsACE2) to renal organoids suppressed viral infection and attenuated the spread of the coronavirus (Fig. 12C) [155].

## 4 Concluding remarks

In this review, we have discussed the current advances in organoid co-cultures for studying host-microbe interactions. Organoid models more accurately mimic the human *in vivo* environment than previous conventional culture systems, offering innovative and effective *in vitro* approaches. Therefore, organoid co-cultures with pathogens or commensals, such as *Salmonella*, *C. difficile* and viruses, have contributed to a better understanding of previously inaccessible interactions between tissues/cells and microbes [156,157]. However, the development of organoids co-cultured with archaea, fungi, and protists are still relatively elusive, compared to the counterpart of bacteria and viruses; organoids-based co-culture studies on a wider range of microbe species should be conducted. In addition, since most of the current microbe-co-cultured organoids research focused on pathophysiological studies, it is necessary to understand the physiological mechanism such as immunity, metabolism, and hormone secretion in the human body and the interaction with symbiotic bacteria through the organoid symbiosis model. Further, the co-culture of organoids with helminth such as *Nippostrongylus brasiliensism* and *Trichinella spirails* can derive an *in vitro* model for parasitism [158,159].

When co-culturing organoids and microbes, a dedicate medium recipe to address the requirement of both is necessary to establish the microbe-co-cultured organoids due to the difference in optimal medium composition for growth and maintenance between two counterparts, in addition to an adequate selection of the co-culture architecture for providing an environment similar to the microenvironment in the corresponding organs *in vivo* [29]. For example, most commensal bacteria in intestine and colon are facultative or obligate anaerobes and can only survive in an environment with minimal oxygen [[160], [161], [162]]. In order to provide specific culture conditions for these microbes, a culture system equipped with a hypoxia gradient could make it possible to reproduce the oxygen gradient *in vivo* [48]. In addition, the control for the number of microbes per single organoid and the timing of microbe inoculation to organoids could enable to obtain more uniform results from microbes-co-cultured organoids system-based applications.

In addition to the studies of the organs mentioned above [35,43,64,99,128,140,152,163,164], co-cultured organoids for other organs, including the esophagus, eye, and blood vessel are increasingly being studied [155,[165], [166], [167]]. Nevertheless, a reconceptualization of the principles of organoid design is required to maximize the applicability of the organoid model [168]. In general, organoids are mostly composed of epithelial and mesenchymal cells, which allows the advantageous feature in obtaining data less relevant from the disturbance or interaction of surrounding cells. However, though organoids grown *in vitro* are more physiologically similar to human than 2D cell cultures, they exhibit fetal-level maturity and function acceptably at various stages of development in physiological studies [169,170]; they also failed to recapitulate *in vivo* tissue condition and organ systems due to a lack of immune cells and vascular structures [170]. Moreover, access to the supply and demand of primary tissues obtained through surgery or biopsy has been becoming increasingly difficult, restricting practical applications of human tissue-derived organoids. Currently available commercial induced pluripotent stem cells not only take a long time to generate mature organoids, but also use enormous media and growth factors; more basic research to identify the optimal conditions for organoid formation is necessary to solve these problems. Meanwhile, the complex and unclear composition of Basement Membrane Extract (BME), a tumor-derived ECM material used for the culture of most organoids, can lead to uncertainty and errors in reproducibility in cell biology [171]. Several types of biopolymers, such as collagen, hyaluronic acid, and poly (ethylene glycol), have been utilized as hydrogel formulations to replace the BME, with the aim of reproducibly generating organoids [[171], [172], [173], [174]]. Nevertheless, more advanced engineering approaches for the polymeric hydrogels for controlling the stiffness, cell viability and bioactivity of the environment required for microbial-host growth is highly needed. Despite these limitations, organoids have been established as standard tools for studying host-microbial interactions and their physiology/pathology [175]. Based on advances in organoid techniques, such as organoids-on-a-chip [176]and assembloids [177], organoids could become an unprecedented model for improving human health in the future.

## Credit author statement

**Min Beom Kim**: Conceptualization, Methodology, Investigation, Writing – original draft, Writing – review & editing. **Soonho Hwangbo**: Writing – review & editing. **Sung Ho Jang**: Writing – review & editing. **Yun Kee Jo**: Conceptualization, Writing – review & editing, Supervision.

## Declaration of competing interest

The authors declare that they have no known competing financial interests or personal relationships that could have appeared to influence the work reported in this paper.
